# COVID-19 Response in Thailand and Its Implications on Future Preparedness

**DOI:** 10.3390/ijerph18031089

**Published:** 2021-01-26

**Authors:** Wijitbusaba Marome, Rajib Shaw

**Affiliations:** 1 Research Unit in Urban Futures and Policy and Faculty of Architecture and Planning, Thammasat University, 99, Khlong Nueng, Khlong Luang District, Pathum Thani 12120, Thailand; wijitbusaba@ap.tu.ac.th; 2 Graduate School of Media and Governance, Keio University, Endo 5322, Fujisawa 252-0882, Japan

**Keywords:** COVID-19, Thailand health response, Sendai framework, inclusive recovery, HEDRM

## Abstract

Thailand has been affected by COVID-19, like other countries in the Asian region at an early stage, and the first case was reported as early as mid-January 2020. Thailand’s response to the COVID-19 pandemic has been guided by the “Integrated Plan for Multilateral Cooperation for Safety and Mitigation of COVID-19”. This paper analyses the health resources in the country and focuses on the response through community-level public health system and legislative measures. The paper draws some lessons on future preparedness, especially with respect to the four priorities of Sendai Framework for Disaster Risk Reduction. At the end, the paper puts some key learning for future preparedness. While Thailand’s response to COVID-19 has been effective in limiting the spread of the disease, it falls short at being able to address the multiple dimensions of the crisis such as the economic and social impacts. The socioeconomic sectors have been hardest hit, with significant impact on tourism sectors. Sociopolitical system also plays an important role in governance and decision-making for pandemic responses. The analysis suggests that one opportunity for enhancing resilience in Thailand is to strive for more multilevel governance that engages with various stakeholders and to support grassroots and community-level networks. The COVID-19 pandemic recovery is a chance to recover better while leaving no one behind. An inclusive long-term recovery plan for the various impacted countries needs to take a holistic approach to address existing gaps and work towards a sustainable society. Furthering the Health Emergency Disaster Risk Management (HEDRM) Framework may support a coordinated response across various linked sectors rather than straining one particular sector.

## 1. Introduction

The COVID-19 pandemic is a public health crisis without precedent in living memory, which is testing our collective capacity to respond” (OECD, 2020 [1]). Caused by SARS-CoV-2 (Severe acute respiratory syndrome coronavirus 2), the infectious COVID-19 (Coronavirus disease 2019) was first identified in Wuhan, China December 2019 and spread across the globe. At the time of writing, the pandemic is ongoing and has affected over 227 countries and territories [2]. The impact of COVID-19 in different countries has varied significantly due to factors including but not limited to governmental response, demographics, and healthcare infrastructure.

This paper focuses on the experience of COVID-19 pandemic in Thailand. As a popular destination for Chinese tourists, it was in Thailand that the first COVID-19 patient outside of China was identified on 13 January 2020. At the end of the same month, the first case of domestic transmission and the 16th patient in Thailand was identified. The number of COVID-19 patients remained relatively low until there was a rise in domestic infections in mid-March. A key event was a boxing match at the Lumpinee Boxing Stadium, which took place on 6 March 2020 despite a ban on large gatherings that was put in place days before. It is estimated that 143 COVID-19 cases were directly linked to the boxing match [3]. In the days following the event, the number of new infections shot up to over a hundred per day.

Thailand’s response to the COVID-19 pandemic has been guided by the “Integrated Plan for Multilateral Cooperation for Safety and Mitigation of COVID-19”, which was drafted by the Ministry of Public Health for the following objectives: (1) Reducing the chances of the virus transmission into Thailand, (2) Everyone in Thailand and Thai people abroad are safe from COVID-19, (3) Mitigating the health, economic, social impacts and increasing national security. The Thai government provided daily updates on COVID-19 infections in Thailand via television and used the media to inform the citizens of new restrictions, safety precautions, and any other official news regarding the pandemic. As of September 2020, the number of COVID-19 cases and new domestic infections in Thailand remains relatively low. Thailand’s ability to contain the spread of COVID-19 thus far has been attributed to factors such as the country’s past experiences with similar epidemics, the citizen’s readiness to wear face masks, a culture of hygiene, and successful public health campaigns [4].

Thailand is often considered as a good practice to reduce the infection rate at an early stage [4]. However, there are little holistic analysis on this. Therefore, this paper aims to provide a short yet comprehensive overview of the responses to COVID-19 in Thailand and to examine the extent to which the measures taken are in line with the guiding principles of Sendai Framework for Disaster Risk Reduction 2015–2030 of the United Nations Office for Disaster Risk Reduction (UNDRR). The Sendai Framework is a landmark document in the domain of disaster risk reduction, which also addresses the challenges brought about by COVID-19, as the framework categorizes epidemics and pandemics as a biological hazard. The case study presented by this paper can be used for comparative analysis of national responses to COVID-19. The findings of this paper bring to light how Thailand can become more resilient to public health crises such as COVID-19 and can therefore be useful not only for academics but also for policymakers and civil society actors. Here, resilience is to be understood as “the ability of a system, community or society exposed to hazards to resist, absorb, accommodate to and recover from the effects of a hazard in a timely and efficient manner, including through the preservation and restoration of its essential basic structures and functions.” [5]. It is argued that the resilience of the country and communities depend on the inherent governance and stakeholder participation, which is linked to four priorities of Sendai Framework.

## 2. Methods

This paper examines how Thailand responded to the COVID-19 pandemic, specifically focusing on the public health and legislative measures. The core of the current analysis lies on the national government policy analysis, supplemented by document reviews and analysis. Information about the measures taken is derived mainly a thorough review of daily governmental announcements about COVID-19, which happen via national television as well as through governmental websites and social media accounts, most notably the website of the Department of Disease Control of the Ministry of Public Health. News articles reporting about COVID-19 were selected from Google searches made with relevant keywords and then analyzed to establish a better understanding of how COVID-19 and the response measures have impacted the public. While governmental announcements and decrees are rarely translated from Thai, English-language articles published by were prioritized to make the cited sources accessible to non-Thai speakers.

The relevant plans and measures taken are critically analyzed through the lens of the Sendai Framework for Disaster Risk Reduction to highlight the robustness and shortcomings of Thailand’s approach with regards to resilience. The analysis is mainly based on: (1) public health capacity and response, and (2) legislative measures. Public health capacities are mainly evaluated based on three issues: (1) health resources in the country, (2) COVID-19 testing, and (3) community based public health initiatives. On the other hand, the legislative measures are analyzed based on Emergency Decree and Disaster Mitigation and Prevention Act. Both the analyses then linked to the four priority areas of Sendai Framework. Sendai Framework.

Sendai framework has four priority areas: (1) understanding disaster risks, (2) strengthen disaster risk governance, (3) investing in disaster risk reduction, and (4) enhancing disaster preparedness for effective response. The Sendai Framework reinforces the scope of disaster risk management by expanding beyond natural hazards to include biological hazards such as epidemic- and pandemic diseases. The Sendai Framework also places strong emphasis on the need to build resilient health systems through the integration of disaster risk management into the provision of health care at all levels and, in particular, “to enhance cooperation between health authorities and other relevant stakeholders to strengthen country capacity for disaster risk management for health.”

## 3. Results

### 3.1. Public Health Capacity and Response

#### 3.1.1. Health Resources in Thailand

While 80% of COVID-19 patients are able to recover without medical treatments [6], many patients develop severe symptoms and may require hospitalization and even intensive care. Patients with lung damage may require the use of non-invasive mechanical ventilators, depending on the severity of the symptoms, to help them breathe. Additionally, hospitals need to have negative pressure isolation rooms and a sufficient amount of personal protective equipment (PPE) to help prevent the spread of the virus within the hospital. Health resources, which refers to “the means available for the operation of health systems, including human resources, facilities, equipment and supplies, financial funds, and knowledge” [7] are therefore critical to the mitigation of the impacts of COVID-19 and prevention of loss of life.

Hospitals in Thailand are divided into three categories, namely (1) public hospitals operated by the Ministry of Public Health, (2) public hospitals operated by other entities (e.g., Medical Service Department of Bangkok Metropolitan Administration, Ministry of Education, Royal Thai Army, Thai Red Cross) and (3) private hospitals. The majority of hospitals in Thailand are public hospitals operated by the Ministry of Public Health, which can be further categorized into central, regional, and community hospitals. According to the Strategy and Planning Division of the Thai Ministry of Health [8], in 2019 there was a total of 158,026 hospital beds at 1370 hospitals across Thailand. This means that Thailand has 2.2 hospital beds per 1000 citizens. To put things into perspective the OECD data shows that the average number of beds per 1000 citizens of its member countries in 2017 was 4.7, with the highest rates being 13.1 and 12.3 beds per 1000 people in Japan and South Korea, respectively [9].

Since 2002, Thailand offers a comprehensive insurance scheme to all its citizens. This scheme was first introduced in 2001 and originally required beneficiaries to contribute a co-payment of 30 baht for medical treatments. The co-pay has since been abolished, and the scheme is now colloquially known as the “gold card” scheme. Universal healthcare is also made available via a civil servant insurance scheme and a private sector employee social security scheme. Under these schemes, 99.5% of the population of Thailand is eligible for healthcare coverage [10].

#### 3.1.2. COVID-19 Testing and Treatment

On 21 March 2020, Thai citizens were told by the government that they should call the hotline of the Department of Disease Control or to go to the hospital that they are registered at to get tested for COVID-19 if they fall under the following criteria:

1. Having had a fever of over 37.5 °C with one or more respiratory symptoms (cough, runny nose, sore throat, rapid breathing or breathing difficulties), in addition to one or more risk factors14 days before the onset of the symptoms.
  (a) Travelling to or from a country or residing in an area with an ongoing COVID-19 outbreak;
  (b) Professional who have had close contact with tourists from areas with ongoing COVID-19 outbreaks;
  (c) Having had close contact or high-risk exposure to confirmed COVID-19 patients in accordance with surveillance and investigation guidelines;
  (d) Being a medical or public health personnel who has come into contact with a suspected or confirmed COVID-19 patient or to the bodily fluids of a suspected or confirmed COVID-19 patient without appropriate protective equipment;
  (e) Having been to a densely populated area at the same time as a confirmed COVID-19 patient, in accordance with the announcement of the provincial infectious disease committee.
2. Pneumonia patients who:
  (a) Has had of close contact with a confirmed COVID-19 patient;
  (b) Is a medical personnel;
  (c) Is experiencing pneumonia without an identifiable cause and who is not recovering within 48–72 h of treatment;
  (d) Exhibits pneumonia characteristics that are consistent with COVID-19.
3. Persons who are suspected to be part of an infection cluster, which is defined as:
  (a) Three or more medical personnel of the same department testing positive for COVID-19 within one week. For smaller establishments e.g., clinics, the criteria used is 3 or more personnel of the establishment;
  (b) For those who are not healthcare workers, five or more infections from the same place within one week with an epidemiological link [11].

Those who fall under any of these criteria can get tested for COVID-19 for free. If they have been infected with the virus, they will also receive free treatments. Those who do not fall under any of these criteria may nevertheless get tested for COVID-19 but at their own cost. In public hospitals, COVID-19 tests cost 2500 to 9900 THB. In private hospitals, the test costs 5000 to 10,500 THB. Under the universal healthcare scheme, confirmed COVID-19 patients will receive free medical treatments according to the procedures of the Ministry of Public Health. Table 1 shows the country data of COVID-19 infection.

#### 3.1.3. Community-Level Public Health Initiatives

In Thailand, the village health volunteers (VHVs) scheme is central to the National Primary Health Care program’s effort to be able to reach more people at a low cost [12]. VHVs are individuals chosen by villagers to receive basic medical training according to the requirements of the Ministry of Public Health in order to help inform and support public health in their community. The initiative started in 1978, and there are now over a million VHVs all over the country. Tasked with the goal of controlling and preventing the spread of diseases, VHVs were influential in mitigating the impacts of the SARS and avian flu outbreaks in Thailand [13]. During the COVID-19 pandemic, VHVs played an important role in preventing the spread of the diseases by going door to door to inform people about COVID-19 symptoms and to screen the residents’ COVID-19 risks by asking them about their travel history and symptoms. They also reported COVID-19 related data to the provincial health office. The VHVs enabled the public to be informed about COVID-19 as well as collected data that are essential for public health decision-making. This is essential to effective COVID-19 response, as demonstrated by a study on how of how China responded to the epidemic [14].

### 3.2. Legislative Measures

On 25 March 2020, the Thai government announced a state of emergency. This allows them to operate according to the Emergency Decree on Public Administration in Emergency Situation, B.E. 2548 (2005) which grants the Prime Minister more decision-making power as well as the ability to impose certain restrictions. The most important clause of the Emergency Decree is Section 9, which explicitly grants the Prime Minister the power to issue regulations to prohibit or restrict personal movements, assemblies, fear-mongering or misleading communications, usage of routes and buildings, as well as to implement evacuations or preventing entry to designated areas. These measures allow the Thai Government to implement restrictions that go beyond what is permitted under the Disaster Prevention and Mitigation Act B.E. 2550, which includes the power to quarantine individuals, to inspect residences, and to restrict the use of routes and vehicles. As of September 2020, the Emergency decree has been extended 5 times and remains active. This has raised some criticisms from those who believe that it is unnecessary for the government to maintain a state of emergency due to a very low rate of domestic COVID-19 infections in Thailand.

One of the first actions of the Prime Minister was the creation of the Center for COVID-19 Situation Administration (CCSA) and an executive committee comprising of ministers and heads of departments that will report directly to the Office of the Prime Minister. They are tasked with implementing policies according to the Communicable Diseases Act B.E. 2558, Disaster Prevention and Mitigation Act B.E. 2550, and the State Administration Act B.E. 2534 (Office of the Prime Minister, 2020). The Thai government has issued numerous announcements of different restrictions such as the closure of certain places and halting some services in order to prevent the spread of COVID-19. Each province also had their own set of restrictions that are issued by the provincial governors. The provincial-level restrictions follow the provisions of the Communicable Diseases Act B.E. 2558 [15].

### 3.3. Analyzing Thailand’s Response to COVID-19 through Sendai Framework

Adopted by UN member states in 2015, the Sendai Framework for Disaster Risk Reduction (2015–2030) is a document that provides guidance on disaster risk reduction. The Thai Department of Disaster Prevention and Mitigation includes epidemics as one of the disasters that they handle, which is appropriate considering the definition of a disaster being “A serious disruption of the functioning of a community or a society at any scale due to hazardous events interacting with conditions of exposure, vulnerability and capacity, leading to one or more of the following: human, material, economic and environmental losses and impacts” [16].

Thailand’s response to the COVID-19 pandemic can be analyzed through the four priorities of the Sendai Framework, which serve as guiding principles for robust and comprehensive disaster risk reduction. Effective and relevant risk management of biological hazards changes with the advancement of science, clinical medicine and public health practices and policy. The latest technical guidelines and research findings to support planning may be found in WHO’s Health-Emergency Disaster Risk Management (Health-EDRM). Although the Sendai Framework strongly focuses on biological hazards and its risk mitigation approaches, there is hardly any country analysis on this.

#### 3.3.1. Understanding Disaster Risk

The Sendai Framework highlights how policies and actions should be based on a thorough understanding of disaster risk. The Thai Disaster Prevention and Mitigation Act B.E. 2550 recognizes epidemics as disasters, so the Department of Disaster Prevention and Mitigation has been involved in the handling of the COVID-19 pandemic. Thailand’s understanding of COVID-19′s various risks is supported by how the country’s previous experiences with respiratory diseases outbreaks such as SARS, avian flu, and MERS. The Ministry of Public Health was at the forefront of the country’s COVID-19 related decision-making and planning. The Ministry oversaw measures such as helping medical facilities around the country prepare for COVID-19 testing and treatment as well as involving VHVs to help the authorities to have a better understanding of the spread of COVID-19, which allows science-based decisions to be made. The Thai government also introduced the contact tracing applications that provide information to the Department of Disease control. However, the applications have been criticized for privacy issues [17].

On the other hand, socioeconomic risks of the pandemic seemed to have been overlooked, as suggested by the government’s limited capacity for providing assistance to vulnerable populations. The Thai government has also been criticized for its insistence to maintain the emergency situation and closed borders despite having had zero cases of domestic infections for over 100 days up until 4 September 2020. Indeed, it is a challenge for governments to determine the optimal stringency of their pandemic measures that would allow them to effectively contain the outbreak without inflicting too much economic damage. Ashraf (2020a) [18] found that “announcements regarding the implementation of social distancing measures by governments have dual, a direct negative and an indirect positive, effect on stock market returns.” While the announcements of social distancing measures “result in negative stock market returns due to their expected adverse impact on economic activity”, they also “lead to positive market returns through the channel of reduction in COVID-19 confirmed cases.” As a country that relies heavily on tourism and from exporting goods, Thailand suffered severe economic impacts due to COVID-19. The Asian Development Bank (ADB) estimated a contraction of −8.0% in the Thai GDP, and that Thai exports of goods and services contracted by 17.6% (in US dollars terms) [19]. Many businesses suffered directly and indirectly from government measures such as border closure and COVID-19 restrictions on restaurants and other services that were introduced during the first and second wave of infections in Thailand. As a result, many people in Thailand lost their income completely or partially. This was especially devastating for day laborer, informal workers, and the urban poor, who also struggled to receive government assistance for reasons such as the lack of access to digital services or difficulties in fulfilling bureaucratic requirements.

For biological hazards, a comprehensive multi-hazard and multi-sectoral National Risk Assessment (NRA) needs to be conducted. The assessment should include exposure, vulnerability, and capacity analyses as part of an integrated policy approach. The cascading effect of different disasters should also be considered, keeping in mind a systemic risk approach. A health risk assessment needs to be an integral component of the risk assessment whenever risk informed public health management is required. For responding to an epidemic or pandemic, an early stage risk assessment and scenario planning incorporating the impacts on different sectors would be required. Real time location-based risk maps should be maintained for enhanced coordination among the different actors. Data sharing and big data analysis also becomes crucial for this step.

Capacity and systems development for integrated risk assessment at the national to a local level is important. Disciplinary divergence (wherever possible) and multi-disciplinary collaboration are key to producing an integrated risk assessment. Higher education/research capacities need to be strengthened, and trans-disciplinary to multi-disciplinary innovative research should be promoted.

#### 3.3.2. Strengthening Disaster Risk Governance to Manage Disaster Risk

For disasters to be managed effectively and efficiently, there should be “Clear vision, plans, competence, guidance and coordination within and across sectors, as well as participation of relevant stakeholders” [19]. Thailand’s COVID-19 response has been guided by the Emergency Situation Decree and was largely led by the Prime Minister via the Center for COVID-19 Situation Administration. However, there is a degree of decentralization as provincial governors were responsible for introducing restrictions against the spread of COVID-19. On 25 January 2020, the Department of Disaster Prevention and Mitigation, which falls under the Ministry of Interior, issued an announcement to all provincial governors to prepare and plan for the spread of COVID-19 in their jurisdiction. The announcement urged the governors to be alert, to make COVID-19 containment plans as well as necessary preparations, and to report any confirmed cases to the Department. On 20 March, the “Integrated Plan for Multilateral Cooperation for Safety and Mitigation of COVID-19” was published. This plan is comprehensive and assigns keys responsibilities to the relevant ministries and departments. The provincial governors were also told to align the local COVID-19 strategies with this plan.

It is important to identify synergies between the health emergency and disaster risk management regulations, and where applicable, review laws specific to epidemics and pandemics to determine implications for disaster risk management. The revision of regulations or legislation related to disaster risk management should be considered to enhance the scope to include biological hazards. The relevant policies and plans also need to be customized.

Science-based, data-centric decision-making is considered important for early identification of hotspots, to provide policy makers with the appropriate advice, and to address collateral hazards. Inclusion of a multi-disciplinary scientific community in national platforms for DRR is important to ensure different perspectives are brought in for decision-making. This will aid in the development of an integrated risk assessment, scenario planning, forecasts of the spread of the epidemic, etc.

#### 3.3.3. Investing in Disaster Risk Reduction for Resilience

Effective disaster risk reduction requires investments for enhancing the resilience of people, their assets, and their environment. Investments include financial and non-financial resources, which can be used for both structural and non-structural measures. In the context of COVID-19, a country’s response is heavily influenced by the investments that have been made both before and during the pandemic not only in public health but also in other domains such as social welfare. Thailand’s experience with COVID-19 highlights the importance of the country’s past investments into the national public health system and to the VHV scheme. The experience also showed how the lack of social safety nets undermines Thailand’s resilience, especially in times of crisis. The government initially struggled to provide the 5000 THB relief to low-income citizens whose livelihood were affected by the pandemic, which contributed to a spike in financially driven suicide attempts in May [20].

As a response to the COVID-19 pandemic, the Thai government unlocked a 2.2 trillion THB (USD 61 billion) relief package that is primarily aimed at providing financial assistance to individuals and SMEs [21,22]. This includes providing financial aid to vulnerable populations, reducing the cost of living by subsidizing natural gas costs and introducing tax reduction, exemption, and delays. However, there is a lack of clarity in how this budget will be spent exactly and there is currently no mention of using it for investments for disaster risk reduction. Through their case study of Japan where governmental financial stimulus was also directed to resilience planning and development, DeWit et al. [23] highlight the importance of a holistic approach to disaster risk reduction by emphasizing that responses to the COVID-19 pandemic should also promote resilience and sustainability in a wider sense.

Investment varies based on the socioeconomic nature of the country. In recent analysis of more than 80 countries socio economic data, Ashraf (2020b) [24] concluded that a stringent lockdown at initial stage, a better social distancing policy and generous economic/fiscal boosting helps the middle to lower income countries to address the socioeconomic issues in pandemic, as well as reduce the number of deaths. In Thailand, the tourism sector plays an important role, which is hardly hit by the pandemic. Farzanegan et al., (2020) [25] concluded that international tourism is highly impacted, which is the same case as Thailand. Thus, investing in boosting domestic travel and tourism to cope with the immediate impact becomes very important. This is also the case of “Go To Travel” campaign in Japan.

Epidemics and pandemics affect wide sectors of society and put both lives and livelihoods at risk, which also impedes development. Fiscal boosting is an important tool for enhancing not only economic recovery, but through a proper social protection measures, can also enhance social safety nets. Existing tools and methods for registering the most vulnerable groups should be adapted to be quickly adjusted and used to identify priority groups for support.

Keeping with a whole-of-society approach, public-private partnerships and business-to-business cooperation are important elements to ensure the continuity of supply chains. This assumes higher significance in the context of supporting small and medium enterprises (SMEs), including those which operate in the informal sector and hence are often left out of social and economic assistance packages.

#### 3.3.4. Enhancing Disaster Preparedness for Effective Response and to “Build Back Better” in Recovery, Rehabilitation, and Reconstruction

According to the Sendai Framework, “Disasters have demonstrated that the recovery, rehabilitation, and reconstruction phase, which needs to be prepared ahead of a disaster, is a critical opportunity to “Build Back Better”, including through integrating disaster risk reduction into development measures, making nations and communities resilient to disasters” [18]. It is unclear whether the Thai government has started planning for post-pandemic actions, and no recovery plan or business continuity plan has been announced as of September 2020. There has also been no announcements business continuity planning. As such, there is currently no known plans for the Thai government to use the COVID-19 budget for sustainable development or for enhancing resilience against public health or environmental hazards.

Early warning is key to responding to any type of hazard, and biological hazards are no exception. A proper early warning system for biological hazards can be developed only when there is a robust public health system in place, which detects any biological hazards before outbreaks occur. This issue needs to be incorporated in development planning as well. Like with natural hazards, a key to early warning is end-to-end communication, where last mile communication is crucial. It is also important that the biological hazard early warning system be integrated into the existing multi-hazard early warning system, which usually focus on natural hazards.

Since epidemics and pandemics are often long lasting, proper business continuity planning for core impacted sectors/ministries is critical. These plans should be developed in advance or at an early stage of the event. Moreover, emergency operation centers should be optimized. Where possible, protocols should be adapted based on lessons from previous disasters while integrating the particularities of the biohazard. Crisis leadership in both the public and private sectors are important. As the situation changes over time, it will be important have an adaptive strategy that can synchronize with the scenario planning.

While most epidemic and pandemic responses focus on impact management, it is equally important to look at root causes and enhance impact reduction. Risk reduction approaches need to be the core of the response mechanism, as well preventative risk reduction in non-emergency decision-making and investment. Volunteers, civil society organizations, and structures at the decentralized level that are working directly on awareness raising and response should be trained regularly to work under the conditions of epidemics and pandemics to ensure their safety and continuity of operations. Lessons from past biological hazards are needed to be taken into account and capacity might need to be strengthened to introduce new institutional arrangements to transform the risk profile.

## 4. Conclusions

While Thailand’s response to COVID-19 has been quite effective in limiting the spread of the disease, it falls short at being able to address the multiple dimensions of the crisis, such as the economic and social impacts. The Sendai Framework for Disaster Reduction helps highlight how Thailand’s approach to COVID-19 does not comprehensively address the issue of resilience—there are limited considerations given to building resilience against climate change or other epidemics. Thailand’s success in containing COVID-19 can be partly attributed to its public health system and to VHVs. VHVs, in turn, benefitted from collaboration with local community leaders and civil society organizations. This suggests that one opportunity for enhancing resilience in Thailand is to strive for more multilevel governance that engages with various stakeholders and to support grassroots and community-level networks.

It can be noted that the Thai approach to COVID-19 response is not very holistic as little consideration has been given to enhancing the country’s resilience to such pandemics as well as to ‘build back better’ in a more environmentally or socially sustainable way. The economic cost of border and business closures and strict restrictions in Thailand has been immense. Businesses in the service and tourism sector were hit the hardest. They are also the sector that employs a large portion of the workforce, who are now financially insecure: Before the COVID-19 pandemic, tourism accounts for 22% of the Thai GDP, and in 2018, one in six jobs in Thailand were in the tourism sector [26]. This sector is highly impacted, along with its link to other formal and informal sectors. The scale of the economic impact of the pandemic as a result of Thailand’s reliance on its tourism sector also highlights the importance of a diversified economy for more resilience. The government’s initial failure to effectively assist low-income and vulnerable populations during the pandemic suggests that they are not included or appropriately prioritized by the government’s COVID-19 risk assessment and planning. However, this seems to have been improved overtime with better governance and decision-making. Although Thailand has been relatively successful at mitigating the public health risks of the COVID-19 pandemic, the impact of the subsequent economic risks and the lack of consideration for building resilience in a broader sense may lead to further vulnerability in the future.

The recovery from past outbreaks, epidemics, and pandemics have shown that an overburdening of the ecosystem services occurred due to increased production for economic gain to make up for losses. Ecosystem services have a more or less constant regeneration cycle, which is disrupted by this sudden surge in demand, thus tipping the threshold of regeneration. Issues of overfishing and clearing of forests could disturb life under water and on land. The increase in production and travel in the aftermath of the health emergency may also increase air and water pollution levels, which might impact the health and wellbeing of people with pre-existing respiratory comorbidities. Further, changes in climatic conditions (temperature, humidity, precipitation, sea level sensitivity) will act as a risk multiplier to other non-communicable and infectious diseases and biological hazards, based on their seasonality and return period. For instance, a decrease in public utility services, like solid waste collection and cleaning of drains may lead to an increase in breading of mosquitoes putting countries identified as dengue hotspots at a higher risk of an outbreak.

The COVID-19 pandemic recovery is a chance to recover better while leaving no one behind. An inclusive long-term recovery plan for the various impacted countries needs to take a holistic approach to address existing gaps and work towards a sustainable society. A biological hazard like the COVID-10 pandemic is an opportunity to strengthen Partnerships under Goal 17 of Sustainable Development Goals (SDGs) to develop warning mechanisms and to reduce gaps in data sharing and accuracy for effective evidence-based policy and decision-making. The role of science and technology and multi-stakeholder partnerships are of importance in such a case. Better global partnerships and effective risk governance need to be brought into the core of preparedness and response for future health emergencies. Furthering the Health Emergency Disaster Risk Management (HEDRM) Framework may support a coordinated response across various linked sectors rather than straining one particular sector.

## Figures and Tables

**Table 1:** COVID-19 data in Thailand as 27th December 2020.

	Number of People
Cumulative cases	6123
Recovered patients	4161
Deaths	60

Data from the Department of Disease Control, Ministry of Health, Government of Thailand.

## Data Availability

Not applicable.
